# Variants of PEAR1 Are Associated With Outcome in Patients With ACS and Stable CAD Undergoing PCI

**DOI:** 10.3389/fphar.2018.00490

**Published:** 2018-05-15

**Authors:** Fabian Stimpfle, Maike Bauer, Dominik Rath, Elke Schaeffeler, Matthias Schwab, Meinrad Gawaz, Stefan Winter, Tobias Geisler

**Affiliations:** ^1^Department of Cardiology and Cardiovascular Medicine, University Hospital of Tübingen, Tübingen, Germany; ^2^Dr. Margarete Fischer-Bosch Institute of Clinical Pharmacology, Stuttgart, Germany; ^3^University of Tübingen, Tübingen, Germany; ^4^Department of Clinical Pharmacology, University Hospital Tübingen, Tübingen, Germany; ^5^Department of Pharmacy and Biochemistry, University Hospital Tübingen, Tübingen, Germany

**Keywords:** PEAR1, rs12566888, rs2768759, rs41273215, rs3737224, rs822442, acute coronary syndrome

## Abstract

**Introduction:** Platelet endothelial aggregation receptor 1 (PEAR1) triggers platelet aggregation and is expressed in platelets and endothelial cells. Genome-wide association studies (GWAS) showed an association between platelet function and single-nucleotide polymorphisms (SNPs) in *PEAR1*.

**Methods:** In 582 consecutive patients with stable coronary artery disease (CAD) or acute coronary syndrome (ACS) scheduled for PCI and treated with ASA and Clopidogrel, Prasugrel, or Ticagrelor, SNP analysis for rs12566888, rs2768759, rs41273215, rs3737224, and rs822442 was performed. During a follow-up period of 365 days after initial PCI, all patients were tracked for a primary endpoint, defined as a combined endpoint consisting of either time to death, myocardial infarction (MI) or ischemic stroke. All cause mortality, MI and ischemic stroke were defined as secondary endpoints.

**Results:** Multivariable Cox model analysis for the primary endpoint revealed a significantly increased risk in homozygous *PEAR1* rs2768759 minor allele carriers (hazard ratio, 3.16; 95% confidence interval, 1.4–7.13, *p* = 0.006). Moreover, *PEAR1* rs12566888 minor allele carriers also showed an increased risk in all patients (hazard ratio, 1.69; 95% confidence interval, 0.87–3.27, *p* = 0.122), which was marginally significant in male patients (hazard ratio, 2.12; 95% confidence interval, 1.02–4.43, *p* = 0.045; *n* = 425).

**Conclusions:** To the best of our knowledge, this is the first study showing that distinct genetic variants of *PEAR1* are associated with cardiovascular prognosis in high risk patients undergoing PCI and treated with dual anti platelet therapy.

## Introduction

Platelet endothelial aggregation receptor 1 (PEAR1) belongs to a unique family of EGF (epidermal growth factor) repeat-containing transmembrane proteins and is highly expressed in platelets and endothelial cells (Nanda et al., 2005). Dextran sulfate triggers platelet aggregation via direct activation of PEAR1 (Vandenbriele et al., 2016).

Several genome-wide association studies (GWAS) showed an association between the single-nucleotide polymorphisms (SNPs) in *PEAR1* and platelet function: rs2768759 and rs12566888 are associated with increased platelet aggregation in healthy individuals and individuals on ASA (Herrera-Galeano et al., 2008; Jones et al., 2009; Johnson et al., 2010; Eicher et al., 2016a). Patients on ASA with angiographically confirmed coronary artery disease (CAD) carrying *PEAR1* rs2768759 SNPs were not at higher risk for death, MI, or stroke (Voora et al., 2011). Sparse data exist regarding clinical outcome after acute coronary syndrome (ACS) and PCI in patients with CAD carrying *PEAR1* SNPs (Voora et al., 2011). The impact of genetic variants of *PEAR1* on platelet response and outcome in patients with CAD undergoing PCI treated with contempory P2Y_12_ inhibitors has insufficiently been studied (Xiang et al., 2013).

## Methods

### Subjects

PEAR1 SNP analysis was performed in 582 consecutive, mostly caucasian patients with stable CAD or ACS receiving PCI. Patients were admitted to the department of cardiology of the University of Tübingen, Germany. All subjects gave written informed consent and the study was approved by the institutional ethics committee (Ethik-Kommission an der Medizinischen Fakultät der Eberhard-Karls-Universität und am Universitätsklinikum Tübingen) (270/2011BO1) and complies with the Declaration of Helsinki and the good clinical practice guidelines (1997; 2001; 2002).

### Genotyping of *PEAR1* variants

Ethylenediaminetetraacetic acid (EDTA) blood samples were collected and genomic DNA was isolated using the QIAmp® DNA Blood Mini Kit System (Qiagen, Hilden, Germany). On the basis of systematic literature search, candidate genetic variants of *PEAR1*, reported to be of functional or clinical importance and representative allele frequency in Caucasians, were selected (Herrera-Galeano et al., 2008; Xiang et al., 2013; Peng et al., 2016; Yao et al., 2016). Thus, the following polymorphisms of *PEAR1* were analyzed: rs12566888, rs2768759, rs41273215, rs3737224, and rs822442.

**Figure 1:**
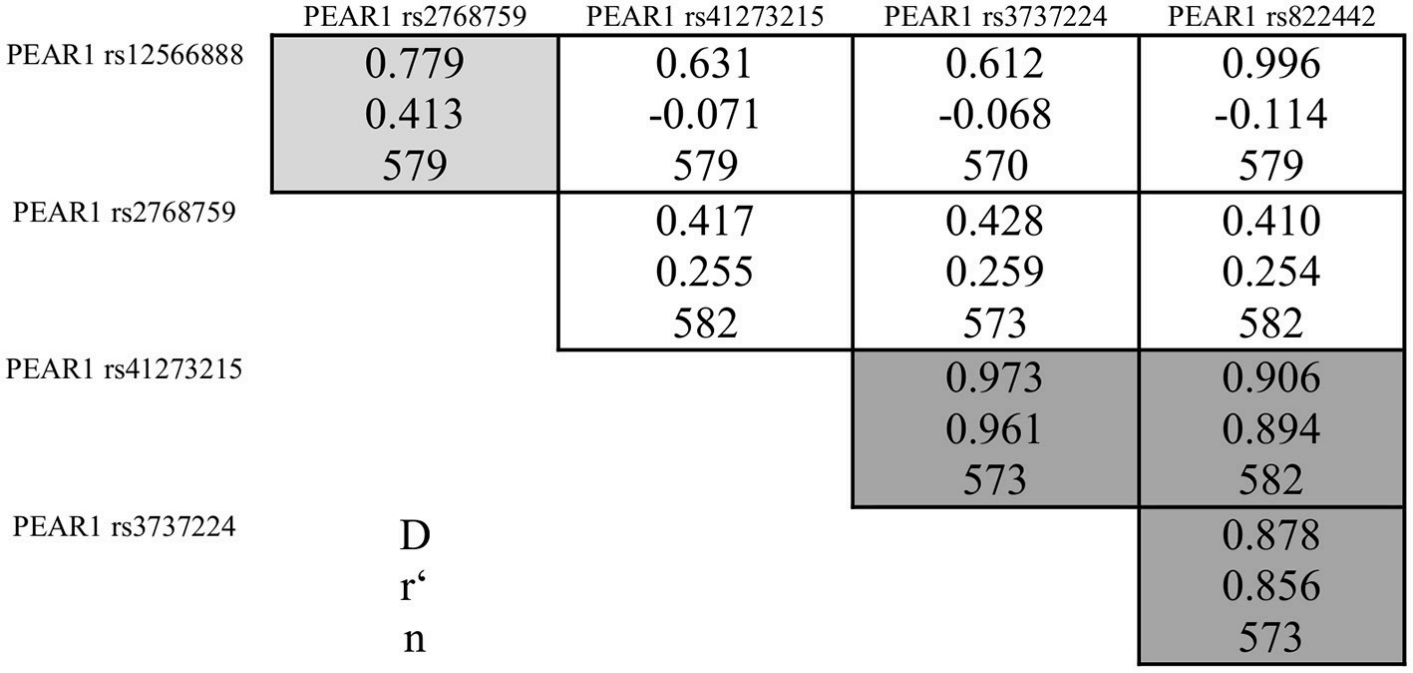
Pairwise linkage disequilibrium between PEAR1 variants rs12566888, rs2768759, rs41273215, rs3737224, and rs822442. Correlation coefficients (*r*) >0.4 are marked in light gray, >0.8 in dark gray.

During the whole genotyping process, study personnel assessing outcome was blinded to the genotype information of the patients. As previously described, genotyping for *PEAR1* variants was performed by matrix-assisted laser desorption/ionization time-of-flight mass spectrometry (MALDI-TOF MS) using the MassARRAY® Compact system (Sequenom, CA, USA) (Schroth et al., 2007) and for quality control, ~10% of samples within each assay were retyped. Details of primers and assays are available upon request. In **Table 2** we provide allele frequencies of *PEAR1* variants in the study cohort. A linkage disequilibrium (LD) map is shown in Figure 1.

### Platelet function analysis

As reported elsewhere (Geisler et al., 2008), blood samples were obtained from venous puncture after loading with P2Y_12_ receptor antagonists and placed in 4.5-ml plastic tubes containing the anticoagulant lepirudin (25 μg/ml; Refludan, Dynabyte, Munich, Germany). Impedance aggregometry (Multiplate® analyzer, ROCHE) was used to measure platelet aggregation induced by adenosine diphosphate (ADP), arachidonic acid (AA), collagen, and thrombin receptor activating peptide (TRAP) on whole blood samples. Aggregation was quantified as area under the curve of aggregation units (AU) (area under the curve = AU × min) (Sibbing et al., 2008, 2009, 2010).

### Follow-up

During a follow-up period of 365 days after initial PCI, all patients were tracked for clinical events including all cause death, myocardial infarction and ischemic stroke. The primary endpoint was defined as a combined endpoint consisting of either first occurrence of death, MI or ischemic stroke. Single events of all-cause mortality, MI and ischemic stroke were defined as secondary endpoints. Follow-up for the primary combined endpoint, performed by telephone interview and/or review of patients' charts on readmission, was terminated after the first occurrence of one of the pre-defined endpoints. Investigators were blinded to the results of laboratory testing.

### Statistical analysis

Statistical analyses were performed using SPSS version 21.0 (SPSS Inc., Chicago, IL, USA) and R-3.3.2 (R Core Team, 2016), including additional packages genetics_1.3.8.1 (Warnes et al., 2013), quantreg_5.33 (Koenker, 2017), SNPassoc_1.9-2 (González et al., 2014), and survival_2.41-3 (Therneau and Grambsch, 2000).

Exact tests of Hardy–Weinberg equilibrium and pairwise linkage disequilibrium between SNPs in our cohort were computed with R-packages SNPassoc and genetics, respectively. In addition, LDlink (https://analysistools.nci.nih.gov/LDlink), a web-based application for exploring population-specific haplotype structure and linkage (Machiela and Chanock, 2015), was used to investigate linkage between SNPs in the European population of the 1000 Genomes Project (EUR, *n* = 1,006; Phase 3, V1; build 144; http://www.internationalgenome.org) (1000 Genomes Project Consortium et al., 2015).

In all subsequent analysis, SNPs were investigated in the dominant (homozygotecarriers (hc) of the major allele vs. minor allele carriers) or recessive (major allele carriers vs. hc carriers of the minor allele) genetic model, as indicated. Associations between SNPs and event rates of primary and secondary endpoints were assessed by Fisher's exact tests. The effects of SNPs on platelet endpoints were investigated by Wilcoxon–Mann–Whitney tests as well as median regression with covariates P2Y_12_ antagonist, reason for admission (stable CAD/type of ACS), arterial hypertension, hyperlipidemia, smoking, diabetes mellitus, medication (ASA, ACE inhibitors, beta blockers, statins), age, gender, and LVEF. Fisher's exact test and Wilcoxon-Mann-Whitney test were applied as appropriate to study the interrelation between SNPs and clinical variables or baseline characteristics. Kaplan–Meier analysis as well as uni- and multivariate Cox models (using the same covariates as in median regression described above) were used to examine the associations between variants and primary and secondary endpoints. All statistical tests were two-sided and significance level was defined as 5%. Unless otherwise stated, *p*-values were not corrected for multiple testing.

## Results

Pairwise linkage disequilibrium analysis showed a strong linkage between rs41273215, rs3737224, and rs822442, while there was only a weak linkage between these SNPs and rs12566888 or rs2768759 (Figure 1). Therefore, only rs12566888, rs2768759, and rs41273215 were further analyzed.

As shown in Table 1, there were no significant or relevant associations between baseline characteristics and all investigated SNPs. Of 579 individuals genotyped for rs12566888, 482 were homozygous for the major allele and 97 were minor allele carriers. Five hundred and forty-three of the Five hundred and eighty-two patients genotyped for rs2768759 were major allele carriers while 39 were homozygous minor allele carriers. In individuals genotyped for rs41273215 461 were homozygous for the major allele and 121 carried the minor allele. As shown in Table 2, major allele frequencies in our cohort, consisting of mostly Caucasian individuals, are perfectly in line with major allele frequencies in the European population of the 1000 Genomes Project. In addition, no considerable deviation from Hardy–Weinberg equilibrium was observed (rs12566888 *p* = 0.439; rs2768759 *p* = 0.914; rs41273215 *p* = 0.0397). Forty of the 582 patients (6.8%) were lost to follow up. The patients lost to follow up did not significantly differ in their baseline characteristics as compared to the group remaining in the study.

**Table 1:** Baseline characteristics.

**Characteristics**	**rs12566888 (*****n*** = **579)**		***p***	**rs2768759 (*****n*** = **582)**		***p***	**rs41273215 (*****n*** = **582)**		***p***
	**hc of major allele (*n* = 482)**	**minor allele carriers (*n* = 97)**		**major allele carriers (*n* = 543)**	**hc of minor allele (*n* = 39)**		**hc of major allele (*n* = 461)**	**minor allele carriers (*n* = 121)**	
No. of males	352 (73%)	71 (73%)	1	394 (73%)	31 (79%)	0.46	339 (74%)	86 (71%)	0.65
Age (years ±*SD*)	69 (±11)	67 (±12)	0.24	69 (±11)	67 (±13)	0.36	68 (±11)	70 (±12)	0.11
**CVRF**									
Arterial hypertension	414 (86%)	76 (78%)	0.063	462 (85%)	31 (79%)	0.35	397 (86%)	96 (79%)	0.064
Hyperlipidemia	295 (62%)	61 (63%)	0.91	336 (62%)	22 (56%)	0.5	293 (64%)	65 (54%)	0.057
Diabetes mellitus	162 (34%)	33 (34%)	1	185 (34%)	11 (28%)	0.49	160 (35%)	36 (30%)	0.33
Smoking	202 (42%)	42 (44%)	0.82	228 (42%)	16 (41%)	1	201 (44%)	43 (36%)	0.12
Family history of CAD	125 (27%)	31 (33%)	0.26	148 (29%)	9 (23%)	0.58	123 (28%)	34 (29%)	0.82
BMI	27 (±7)	27 (±6)	0.73	27 (±7)	28 (±4)	0.6	27 (±7)	27 (±5)	0.56
**CLINICAL FACTORS**									
Atrial fibrillation	103 (21%)	19 (20%)	0.79	115 (21%)	7 (18%)	0.84	234 (51%)	57 (48%)	0.54
LVEF% impairment	239 (50%)	51 (53%)	0.58	270 (50%)	21 (54%)	0.74	1 (±0)	1 (±0)	0.75
Renal function (creatinine ±SD)	1 (±0)	1 (±0)	0.36	1 (±0)	1 (±1)	0.62	186 (41%)	39 (32%)	0.092
Gensini score	184 (39%)	39 (41%)	0.65	210 (39%)	15 (41%)	1	36 (8%)	16 (13%)	0.076
Previous PCI	44 (9%)	7 (7%)	0.69	48 (9%)	4 (11%)	0.77	143 (31%)	29 (24%)	0.15
CABG	141 (29%)	30 (31%)	0.81	162 (30%)	10 (26%)	0.72	234 (51%)	57 (48%)	0.54
Previous MI	103 (21%)	19 (20%)	0.79	115 (21%)	7 (18%)	0.84	1 (±0)	1 (±0)	0.75
**MEDICATION ON ADMISSION**									
Acetyl salicylic a*c*id	284 (59%)	54 (56%)	0.65	317 (58%)	23 (59%)	1	267 (58%)	73 (60%)	0.68
Clopidogrel	63 (13%)	14 (15%)	0.93	72 (13%)	5 (13%)	0.89	65 (14%)	12 (10%)	0.45
Prasugrel	7 (1%)	1 (1%)		8 (1%)	0 (0%)		7 (2%)	1 (1%)	
Ticagrelor	17 (4%)	2 (2%)		18 (3%)	2 (5%)		14 (3%)	6 (5%)	
No P2Y_12_ antagonist	395 (82%)	79 (82%)		444 (82%)	32 (82%)		374 (81%)	102 (84%)	
Oral anticoagulants	42 (9%)	9 (9%)	0.84	49 (9%)	3 (8%)	1	41 (9%)	11 (9%)	1
ACE inhibitors	203 (42%)	39 (41%)	0.82	229 (42%)	15 (38%)	0.74	193 (42%)	51 (42%)	1
AT1-receptor antagonists	96 (20%)	20 (21%)	0.89	107 (20%)	9 (23%)	0.68	88 (19%)	28 (23%)	0.37
Ca-channel inhibitors	101 (21%)	24 (25%)	0.42	116 (21%)	11 (28%)	0.32	101 (22%)	26 (21%)	1
Beta blockers	287 (60%)	62 (65%)	0.42	329 (61%)	22 (56%)	0.61	282 (61%)	69 (57%)	0.4
Statins	237 (49%)	50 (52%)	0.66	271 (50%)	19 (49%)	1	233 (51%)	57 (47%)	0.54
Platelet count^*^	250 (±83)	237 (±72)	0.28	248 (±81)	247 (±79)	0.98	249 (±72)	244 (±109)	0.028
Mean platelet volume^†^	13 (±15)	15 (±22)	0.6	13 (±14)	22 (±33)	0.68	14 (±18)	10 (±1)	0.18
**REASON OF ADMISSION**									
STEMI	50 (10%)	11 (11%)	0.75	56 (10%)	5 (13%)	0.81	50 (11%)	11 (9%)	0.67
NSTEMI	103 (21%)	23 (24%)		116 (21%)	10 (26%)		100 (22%)	26 (21%)	
Unstable CAD	102 (21%)	23 (24%)		118 (22%)	8 (21%)		95 (21%)	31 (26%)	
Stable CAD	227 (47%)	40 (41%)		253 (47%)	16 (41%)		216 (47%)	53 (44%)	

* *Platelet count in platelets/μl*.

† *Mean platelet volume in fl*.

**Table 2:** Major allele frequencies of *Pear1* variants rs12566888, rs2768759 and rs41273215 in the study cohort in comparison to the 1000 genomes project (EUR, *n* = 1,006).

	**Allele**	**MAF**	**HWE *p*-value**	**MAF 1000 Genomes; EUR**
PEAR1 rs12566888	G/T	91.1	0.439	90.8
PEAR1 rs2768759	C/A	74.2	0.914	72.0
PEAR1 rs41273215	C/T	88.5	0.0397	89.7

*MAF, Major allele frequency; HWE, Hardy–Weinberg equilibrium*.

**Figure 2:**
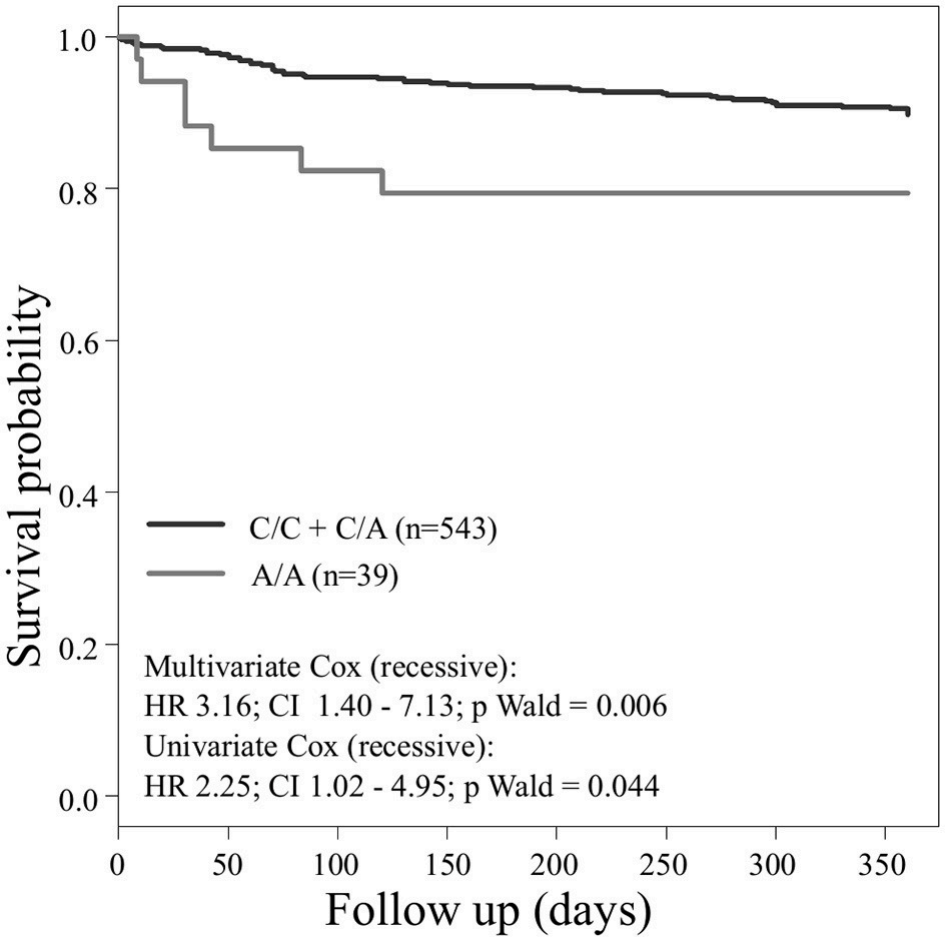
Kaplan–Meier curves showing cumulative survival (combined endpoint all-cause death and/or Ml and/or ischemic stroke) stratified according to *Pear1* rs2768759 carriers of major allele (black) and homozygous carriers of minor allele (gray).

**Figure 3:**
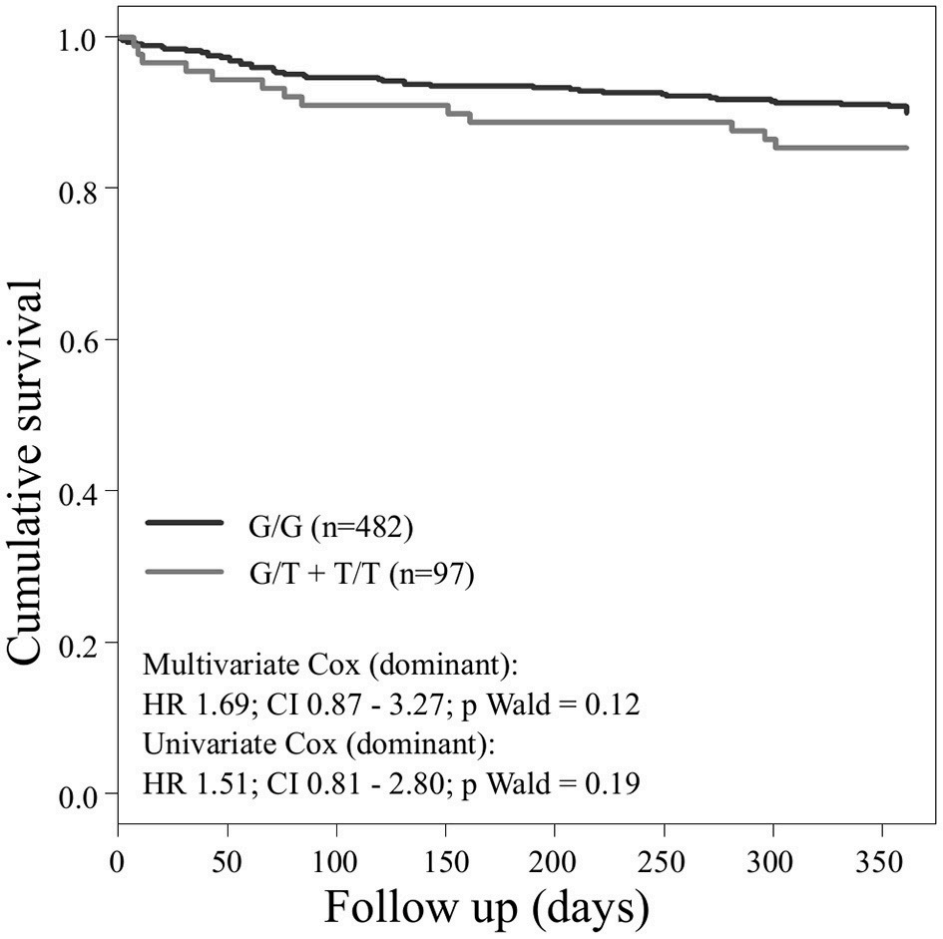
Kaplan–Meier curves showing cumulative survival (combined endpoint all-cause death and/or Ml and/or ischemic stroke) stratified according to *Pear1* rs12566888 homozygous carriers of major allele (black) and carriers of minor allele (gray).

**Figure 4:**
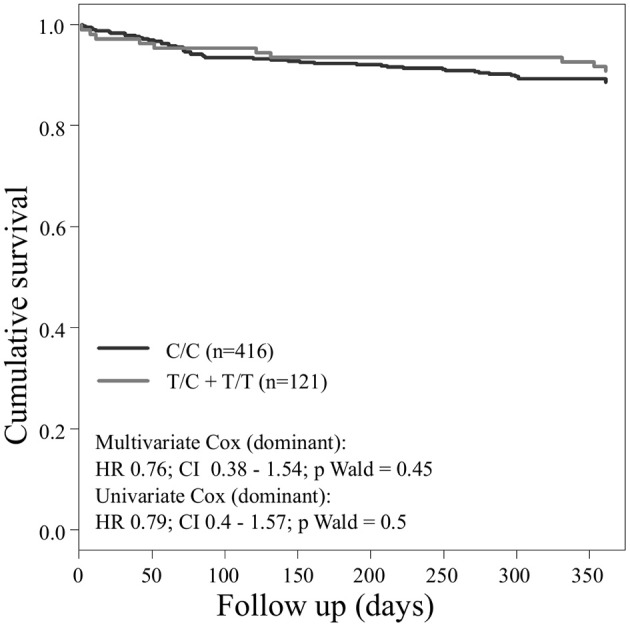
Kaplan–Meier curves showing cumulative survival (combined endpoint all-cause death and/or Ml and/or ischemic stroke) stratified according to *Pear1* rs41273215 homozygous carriers of major allele (black) and carriers of minor allele (gray).

Concerning the primary combined endpoint, multivariate Cox proportional hazard analysis with adjustment for age, gender, LVEF, type of ACS or stable CAD, cardiovascular risk factors such as hypertension, hyperlipoproteinemia, smoking or diabetes, as well as medication (ACE-inhibitors, beta-blockers, statins, and antiplatelet therapy) revealed a significant difference between major and homozygous minor allele carriers of *PEAR1* rs2768759 (*p* = 0.006; Wald test) as shown in Figure 2 and **Table 4**. A similar difference was observed between minor and homozygous major allele carriers of *PEAR1* rs12566888, which however failed to be significant (*p* = 0.122; Wald test, Figure 3). *PEAR1* rs41273215 was not associated with the primary combined endpoint (*p* = 0.45; Wald test, Figure 4).

Secondary endpoints also differed between major and homozygous minor allele carriers of *PEAR1* rs2768759 carriers. Multivariate Cox proportional hazard analysis showed a significant association of this SNP with myocardial infarction (*p* = 0.013; Wald test) and a borderline significant association of rs2768759 and all-cause mortality (*p* = 0.051; Wald test). *PEAR1* rs12566888 and rs41273215 were not significantly associated with secondary endpoints (Table 4). Event and incident rates are shown in Table 3.

**Table 3:** Events and incident rate (IR)/100 person years (PY) in the overall cohort.

**Variable**	**rs12566888 hc of major allele**		**rs12566888 minor allele carriers**		***P* (Fisher's exact test)**
	**Number of events**	**IR/100 PY**	**Number of events**	**IR/100 PY**	
All cause mortality	20	3.525	5	8.6	0.585
Ischemic stroke	7	1.2	2	0.3	0.648
Myocardial infarction	28	4.8	9	1.6	0.175
Combined endpoint	45	7.8	13	2.2	0.194
	**rs2768759 major allele carriers**		**rs2768759 hc of minor allele**		***P*** **(Fisher's exact test)**
All cause mortality	22	3.8	3	0.5	0.202
Ischemic stroke	9	1.5	0		1
Myocardial infarction	33	5.7	5	0.9	0.08
Combined endpoint	52	8.9	7	1.2	0.081
	**rs41273215 hc of major allele**		**rs41273215 minor allele carriers**		**P (Fisher's exact test)**
All cause mortality	22	3.8	3	0.5	0.444
Ischemic stroke	8	1.4	1	0	0.694
Myocardial infarction	31	5.3	7	1.2	1
Combined endpoint	49	8.4	10	1.7	0.608

**Table 4:** Multivariate Cox proportional hazard analysis of the primary combined endpoint.

	Multivariable Cox proportional hazard analysis^*^		
**Endpoint**	**HR**	**95%CI**	***p*-value (Wald test)**
**rs12566888^†^**			
Combined endpoint	1.69	0.87, 3.27	0.122
Myocardial infarction	1.67	0.74, 3.77	0.221
All cause mortality	2.10	0.73, 6.09	0.170
**rs2768759^‡^**			
Combined endpoint	3.16	1.4, 7.13	**0.006**
Myocardial infarction	3.44	1.3, 9.11	**0.013**
All cause mortality	3.62	1.0, 13.12	0.051
**rs41273215^‡^**			
Combined endpoint	0.76	0.38, 1.54	0.451
Myocardial infarction	0.92	0.4, 2.14	0.849
All cause mortality	0.52	0.14, 1.91	0.328

* *With covariates P2Y12 antagonist, reason for admission (stable CAD/type of ACS), arterial hypertension, hyperlipidemia, smoking, diabetes mellitus, medication (ASA, ACE inhibitors, beta blockers, statins), age, gender, and LVEF*.

† *hc of major allele vs. minor allele carriers (dominant model)*.

‡ *Major allele carriers vs. hc of minor allele (recessive model)*.

Platelet volume and count as well as platelet function, measured with different agonists, did not differ significantly between minor and homozygous major allele carriers of rs12566888 or major and homozygous minor allele carriers of rs2768759. Concerning rs41273215, the platelet count was significantly lower in minor allele carriers as compared to and homozygous major allele carriers (median 218,000/μl vs. 243,000/μl, Wilcoxon–Mann–Whitney test *p* = 0.028, median regression *p* = 0.036). Likewise, TRAP induced platelet aggregation indicated a lower platelet reactivity in rs41273215 minor vs. homozygous major allele carriers allele carriers (median 75 vs. 83; Wilcoxon–Mann–Whitney test *p* = 0.0094, median regression *p* = 0.0042). Platelet aggregation induced by ADP, arachidonic acid and collagen under dual antiplatelet therapy (DAT) was not significantly associated with any of the investigated *PEAR1* variants. Platelet parameters and function analysis are shown in Table 5.

**Table 5:** Platelet function analysis.

**rs12566888**	**hc of major allele median**	**minor allele carriers median**	**Difference in location**	**Lower/upper 95% CI**	**WMW test *p*-value**	Median regression *p*-value^*^
Mean platelet volume	9.8	5.9	0.2	−0.6, 1	0.6	0.31
Platelet count	238	226	8	−6, 23	0.28	0.083
**Platelet aggregation test:**						
ADP	34	24	4	0, 9	0.065	0.13
Arachidonic acid	13	12	1	−1, 3	0.33	0.43
TRAP test	82	79	5	−1, 11	0.09	0.15
Collagen test	36	33	4	−5, 15	0.37	0.88
**rs2768759**	**major allele carriers median**	**hc of minor allele median**	**Difference in location**	**Lower/upper 95% CI**	**WMW test** ***p-*****value**	**Median regression** ***p***-value^*^
Mean platelet volume	8.90	9.60	0.2	−0.9, 0.9	0.68	0.056
Platelet count	236	240	0	−26, 26	0.98	0.89
**Platelet aggregation test:**						
ADP	32	24	4	−3, 12	0.25	0.21
Arachidonic acid	13	11.50	2	−1, 5	0.12	0.63
TRAP test	82	75.5	3	−6, 12	0.49	0.14
Collagen test	37	33	6	−6, 24	0.32	0.35
**rs41273215**	**hc of major allele median**	**minor allele carriers median**	**Difference in location**	**Lower/upper 95% CI**	**WMW test** ***p*****-value**	**Median regression** ***p***-value^*^
Mean platelet volume	9.90	9.55	0.3	−0.2, 0.8	0.18	0.12
Platelet count	243	218	14	1, 27	**0.028**	**0.036**
**Platelet aggregation test:**						
ADP	33	26	4	−1, 8	0.1	0.13
Arachidonic acid	13	13	1	−1, 2	0.45	0.3
TRAP test	83	75	7	2, 12	**0.0094**	**0.0042**
Collagen test	37	30	7	−2, 18	0.13	0.73

* *With covariates P2Y_12_ antagonist, reason for admission (stable CAD/type of ACS), arterial hypertension, hyperlipidemia, smoking, diabetes mellitus, medication (ASA, ACE inhibitors, beta blockers, statins), age, gender, and LVEF*.

*WMW, Wilcoxon-Mann-Whitney*.

At discharge, 66% of the patients were treated with clopidogrel, 15% with prasugrel, and 18% with ticagrelor.

## Discussion

Platelet endothelial aggregation receptor 1, which is highly expressed in platelets and endothelial cells, is a membrane tyrosine kinase receptor that mediates platelet activation (Nanda et al., 2005). The gene encoding *PEAR1* is located on the human chromosome 1q23.1 and several SNPs have been described.

Genome-wide association studies (GWAS) showed that *PEAR1* gene variants are associated with greater platelet aggregability (Herrera-Galeano et al., 2008; Johnson et al., 2010; Faraday et al., 2011) and it has been reported, that SNPs in *PEAR1* are associated with an increased risk of myocardial infarction (Lewis et al., 2013). Several studies showed an association between SNPs of *PEAR1* and platelet aggregation in response to different agonists in healthy individuals: The C allele of SNP rs2768759 is associated with increased collagen, epinephrine, and ADP-induced platelet aggregation. This association was even stronger and more consistent after administration of ASA, suggesting a relationship between the C allele and reduced platelet responsiveness to ASA (Herrera-Galeano et al., 2008). Jones et al. have demonstrated that SNP rs41299597 is associated with an increased expression of *PEAR1* in activated platelets (Jones et al., 2009). In a meta-analysis of GWAS Johnson et al. (2010) analyzed platelet aggregation responses to ADP, epinephrine and collagen in 4000 individuals of European ancestry and found *PEAR1* SNP rs12566888 to be genome-wide significant for association with ADP-induced aggregation (*P* = 3.4 × 10^−12^). Eicher et al. performed a large-scale meta-analysis of Exomechip association results for platelet count (PTL) in 157,293 individuals and mean platelet volume (MPV) in 157,293 individuals and showed that *PEAR1* is not only associated with PLT und MPV but also with platelet reactivity (Eicher et al., 2016b). In another study, they found a strong association between rs12566888 in *PEAR1* and ADP (*p* = 1.51 × 10^−7^) and thrombin-induced (*p* = 1.91 × 10^−6^) platelet reactivity in platelet rich plasma (Eicher et al., 2016a). Several studies on genetic variants of *PEAR1* provided evidence that the intronic SNP rs12041331 alters PEAR1 protein expression and platelet function (Faraday et al., 2011; Kauskot et al., 2012; Kunicki et al., 2012; Qayyum et al., 2015) and Western blotting and ELISA confirmed dose-response relation between the number of major G alleles at rs12041331 and expression of PEAR1 protein (Faraday et al., 2011; Izzi et al., 2016). For ADP-induced aggregation, the *PEAR1* minor allele was associated with a decreased response (Kunicki et al., 2012).

PEAR1 is also expressed in endothelial cells (Nanda et al., 2005) and, as data from several studies indicate, SNPs seem to alter endothelial function: The major G allele of rs12041331 is associated with higher *PEAR1* expression than the minor A allele, not only platelets but also in endothelial cells (Izzi et al., 2016). Fisch et al. observed that variation of *PEAR1* significantly determines endothelial function. They found a significant association between rs12041331 and flow-mediated dilation of the brachial artery in 641 individuals. Several genes that play an important role in endothelial function, such as *ANG2, ACVRL1*, or *ENG*, are highly correlated with *PEAR1* expression and endothelial cell migration and angiogenesis are influenced by *PEAR1* (Fisch et al., 2015). An inverse correlation between vascular assembly both *in vitro* and *in vivo* and endothelial PEAR1 expression was reported, identifying *PEAR1* as a novel modifier of neoangiogenesis (Vandenbriele et al., 2015).

In patients on aspirin alone or in combination with clopidogrel, genetic variation in *PEAR1* may affect on-treatment platelet reactivity and be associated with increased risk for cardiovascular events. Genetic variants in *PEAR1* influence platelet aggregation in healthy individuals on ASA (rs2768759, rs12041331) (Herrera-Galeano et al., 2008; Backman et al., 2017) and in aspirin-treated patients with CAD (rs12041331) (Wurtz et al., 2014). Aspirin-treated rs12041331 A-allele carriers in the INVEST-GENES study had significantly increased risk of myocardial infarction compared with GG homozygotes (odds ratio, 2.03; 95% confidence interval, 1.01–4.09; *P* = 0.048). Also, this SNP was strongly associated with response to dual antiplatelet therapy (*P* = 7.66 × 10^−9^) (Lewis et al., 2013). In patients undergoing percutaneous coronary intervention (PCI), A-allele carriers of rs12041331 experienced a cardiovascular event or death more frequently compared with GG homozygotes (hazard ratio, 2.62; 95% confidence interval, 0.96–7.10; *p* = 0.059; and hazard ratio, 3.97; 95% confidence interval, 1.10–14.31; *P* = 0.035, respectively) (Lewis et al., 2013). However, patients carrying *PEAR1* rs2768759 SNPs, diagnosed with angiographically confirmed CAD and treated with aspirin were not at higher risk for death, MI, or stroke (Voora et al., 2011). Yao et al. recently reported, that in Chinese patients with acute myocardial infarction, PEAR1 rs56260937 minor allele predicts adverse ischemic events after PCI (Yao et al., 2017). We performed linkage disequilibrium analyses based on 1000 Genomes data, that schowed rs56260937 is moderately linked to rs41273215 (*r* = 0.55, D′ = 0.74).

In our entire cohort we could not find a significant alteration of on-treatment platelet reactivity in individuals carrying variant alleles of *PEAR1* except for decreased TRAP-induced aggregation in minor carriers of rs41273215. In contrast to our results, Herrera-Galeano et al. found that SNP rs2768759 [A/C] is associated with high on-treatment in individuals with premature CAD on ASA (Herrera-Galeano et al., 2008). An association between rs12566888 in *PEAR1* and ADP and thrombin-induced platelet reactivity was described by Eicher et al. (2016a). Nevertheless, the individuals investigated in these studies were not treated with P2Y_12_ receptor antagonists and did not undergo PCI. Moreover, the cohort of Eicher et al. comprised only men. An additional subgroup analysis of our cohort revealed that rs12566888 was significantly correlated with TRAP test in male (median regression *P* = 0.016) but not in female patients (*P* = 0.78), suggesting a gender-specific effect. In contrast, the associations between rs12566888 and ADP as well as rs2768759 and platelet aggregation remained insignificant when we investigated men and women separately. In addition, MPV and PTL were not associated with these *PEAR1* variants, which is in line with a large-scale meta-analysis performed by Eicher et al. (2016b).

It has been reported, that in patients undergoing PCI and carrying rs12041331 cardiovascular events and death occur more frequently (Lewis et al., 2013). Rs12041331 is strongly linked to rs12566888 in Caucasians as reported by LDlink (Machiela and Chanock, 2015) based on 1000 Genomes data (EUR, *n* = 1,006). In our cohort, rs2768759 was significantly associated with the primary endpoint, consisting of all-cause mortality, MI and ischemic stroke. In individuals homozygous for the minor allele, the primary endpoint occurred with an incidence rate of 8.9/100 PY compared to 1.2/100 PY in major allele carriers. In multivariate Cox proportional hazard regression analysis with adjustment for cardiovascular risk factors we found this difference to be highly significant (*p* = 0.006). This difference could in large parts be attributed to the secondary endpoint MI which occurred with an IR of 5.7/100 PY in individuals homozygous for the minor allele compared to 0.9/100 PY in major allele carriers (Wald test *P* = 0.013). Voora et al. reported that patients carrying *PEAR1* rs2768759 SNPs who were diagnosed with angiographically confirmed CAD and treated with aspirin were not at higher risk for death, MI, or stroke (Sibbing et al., 2010). This is not in line with our findings. However, the individuals investigated by Voora et al. were not treated with P2Y_12_ receptor antagonists and did not undergo PCI.

In our cohort, in patients who were homozygous major allele carriers of rs12566888, the primary endpoint occurred more frequently (IR of 7.8/100 PY vs. 2.2/100 PY in homozygous major allele carriers vs. minor allele carriers) but failed statistical significance in multivariate Cox proportional hazard regression analysis (Wald test *P* = 0.122). A recently published study by Yang et al. could also not replicate previous reports suggesting that SNP rs12566888 in *PEAR1* might be a susceptibility gene for cardiovascular complications in Caucasians (Yang et al., 2017). Nevertheless, the individuals studied by Yang et al. did not undergo PCI like our cohort and the fact that the primary endpoint failed to be significantly associated with rs12566888 in our study collective may also be due to its moderate sample size and possibly gender-specific effects. Indeed, multivariable Cox modeling revealed a marginally significant association between rs12566888 and the combined endpoint in men (Wald test *P* = 0.045), but not in women (*P* = 0.352). In contrast, the effect of rs12566888 on myocardial infarction or all cause mortality was neither significant in the entire cohort nor in the gender subgroups. Of note, the study of Yang et al. and the patients undergoing PCI in Lewis et al. (2013) comprised 48.7 and 60% men, respectively. Altogether, the question whether rs12566888 predicts inferior outcome in patients undergoing PCI warrants further investigation.

Yao et al. showed that in an Asian cohort of patients undergoing PCI, on-treatment platelet reactivity under ASA and Clopidogrel was higher in rs41273215 minor allele carriers (*p* = 0.025) (Yao et al., 2016). In contrast to Yao's findings, on-treatment platelet reactivity was not associated with minor allele T at rs41273215 in our study of patients of Caucasian ancestry. Though, TRAP-induced platelet reactivity was significantly lower in individuals carrying this SNP. Moreover, ADP was marginally significantly lower in male rs41273215 minor allele carriers (median regression *P* = 0.047) but not in females (*P* = 0.14). Jones et al. showed altered platelet reactivity in healthy rs41273215 carriers.

To prove stability of the model we replaced cardiovascular comedications by additional cardiovascular comorbidities including history of myocardial infarction and atrial fibrillation. In summary, most of the associations of PEAR genetic variants with platelet function parameters and clinical outcome remained unchanged.

To conclude, this is to the best of our knowledge the first report of the prognostic impact of selected *PEAR1* SNPs in a cohort of cardiovascular patients with ACS or stable CAD undergoing PCI and treated with contemporary DAPT. We found that rs2768759 predicts adverse outcome regarding the incidence of MI as well as a combined endpoint consisting of all-cause mortality, MI and ischemic stroke. Rs12566888 was only marginally significantly correlated with the combined endpoint in men and rs41273215 was not found to be significantly associated with cardiovascular outcome, possibly due to the moderate sample size of our collective. TRAP-induced aggregation was significantly decreased in minor allele carriers of rs41273215 and in male minor allele carriers of rs12566888. Moreover, there was a strong trend toward lower ADP induced aggregation in male minor allele carriers of rs41273215.

## Limitations

The current study has some limitations. First, it represents a candidate-gene study with all limitations and we cannot rule out influences of other genetic variants not investigated. Our findings have not been validated in an independent cohort or a genome-wide association approach. A sample size calculation based on a log-rank test showed that in order to validate the effect of rs2768759 with 80% power and for a significance level of 5%, an additional cohort with at least *n* = 737 patients would be required (hazard ratio 3.2, event rate 11%). The present study is of moderate sample size and observational character and needs replication in further large scale studies. Our findings also need replication in other ethnic groups as our cohort consists mainly of Caucasian individuals.

## Author contributions

FS: patient selection, statistical analysis, and drafting of the manuscript; MB: patient baseline characteristics and follow-up; DR: critical revision; ES: genotyping and drafting of the manuscript; SW: statistical analysis and drafting of the manuscript; MS: critical revision and funding; MG: critical revision and funding; TG: drafting of the manuscript and funding.

### Conflict of interest statement

The authors declare that the research was conducted in the absence of any commercial or financial relationships that could be construed as a potential conflict of interest.
